# Risk of adverse obstetrical and neonatal outcomes in women consuming recreational drugs during pregnancy

**DOI:** 10.1186/s12884-024-07062-1

**Published:** 2025-04-16

**Authors:** Sreemanjari Kandhasamy, Karine Lepigeon, Stéphanie Baggio, Roulet Céline, Michael Ceulemans, Ursula Winterfeld, Stephen P. Jenkinson, Katyuska Francini, Emeline Maisonneuve, Alice Panchaud

**Affiliations:** 1 Instiute of Primary Health Care (BIHAM), University of Bern, Mittelstrasse 43, 3012 Bern, Switzerland; 2 Graduate School of Health Sciences, University of Bern, Bern, Switzerland; 3 Materno-Fetal and Obstetrics Research Unit, Department “Women-Mother-Child”, Lausanne University Hospital, 1011 Lausanne, Switzerland; 4 Laboratory of Population Health (#PopHealthLab), University of Fribourg, Fribourg, Switzerland; 5 Department of Pharmaceutical and Pharmacological Sciences, KU Leuven, Leuven, Belgium; 6 L-C&Y, KU Leuven Child and Youth Institute, Leuven, Belgium; 7 Department for Health Evidence, Radboud University Medical Centre, Nijmegen, the Netherlands; 8 Swiss Teratogen Information Service, Clinical Pharmacology Service, Lausanne University Hospital and University of Lausanne, Lausanne, Switzerland; 9 Community Pharmacy, Center for Primary Care and Public Health (UNISANTÉ), University of Lausanne, Lausanne, Switzerland; 10 Service of Pharmacy, Lausanne University Hospital and University of Lausanne, 1011 Lausanne, Switzerland

**Keywords:** Pregnancy, Adverse outcomes, Substance use, Cocaine, Opioids

## Abstract

**Background:**

Previously conducted studies have observed an increased risk of adverse maternal and neonatal outcomes with prenatal exposure to cocaine and opioids. However, these studies used drug-free reference groups which did not efficiently control for confounders associated with polysubstance use in pregnancy. Thus, we conducted an observational study to compare the risk of adverse obstetrical and neonatal outcomes in women who consumed cocaine and/or opioids during pregnancy to women who consumed only cannabis in pregnancy.

**Methods:**

This observational study was conducted with data collected from pregnant women followed for addiction from the beginning of their pregnancy until childbirth at the perinatal consultation center Addi-Vie at CHUV Lausanne, Switzerland. Women who reported consuming cocaine, opioids, or both along with or without cannabis were included in the exposed group, while women who reported use of only cannabis during pregnancy were included in the reference group. We assessed for two adverse composite outcomes namely: adverse obstetrical composite outcome (4 outcomes) and adverse neonatal composite outcome (7 outcomes). Weighted logistic regression with weights obtained through inverse probability treatment weighting was conducted. For this analysis, we reported a conditional odds ratio (OR_conditional_) and 95% confidence interval (CI).

**Results:**

We included 177 pregnant women in this study, with 80 included in the exposed group and 97 included in the reference group. In the exposed group, 81.2% of women reported the use of opioids, and 39.9% of women reported the use of cocaine during pregnancy. In this study, prenatal cocaine and/or opioid exposure was associated with reduced odds of adverse obstetrical composite outcomes (OR_conditional_: 0.39, 95% CI: 0.17–0.88) compared to prenatal cannabis use. We also observed that the pregnant women exposed to cocaine and/or opioids during pregnancy were at 3.88 (OR_conditional_: 3.88, 95% CI: 1.23–12.23) times higher odds of experiencing the adverse neonatal composite outcome compared to our reference group.

**Conclusion:**

Prenatal use of cocaine and/or opioids during pregnancy is observed to increase the odds of adverse neonatal composite outcomes. Encouraging substance users to seek antenatal care in earlier stages of pregnancy and targeted treatment approaches through interprofessional collaboration could prevent such adverse outcomes in pregnancy.

**Supplementary Information:**

The online version contains supplementary material available at 10.1186/s12884-024-07062-1.

## Background

Recreational drugs are substances used for non-medical purposes, particularly for their psychoactive nature, and are often believed by the user that the occasional use of these substances would not turn out to be addictive [1]. However, the use of illicit drugs (recreational drugs that are illegal to consume) during pregnancy is a growing public health concern. In the USA, the percentage of women reporting the consumption of illicit drugs during pregnancy increased from 5.9% in 2012 to 7.7% in 2021 [2, 3]. Moreover, in 2020, the prevalence of illicit drug use among pregnant women in Europe and Asia was estimated to be around 5% and 6%, respectively [4]. Pregnant women who use illicit drugs frequently report the use of cocaine, opioids, and cannabis [4].

The consumption of these illicit drugs during pregnancy increases the risk of several adverse maternal and neonatal outcomes. Use of cocaine during pregnancy increases the risk of placental abruption, premature rupture of membranes, preterm birth, intrauterine growth restriction, and small for gestational age (SGA) [5–7]. Prenatal exposure to heroin, an illicit opioid, increases the risk of lower birth weight, reduced birth length, and the diagnosis of neonatal abstinence syndrome (NAS) in infants [8, 9]. A greater risk of adverse obstetrical outcomes such as vaginal bleeding in the third trimester, fetal distress, fetal malpresentation, pre-eclampsia, and neonatal aspiration of meconium was observed in pregnant heroin users [10]. Heroin dependency in pregnancy is treated with opioid agonist therapy with methadone or buprenorphine. Although opioid agonist treatment with buprenorphine in pregnant women had better neonatal outcomes, like increased head circumference, increased birth weight, and shorter duration of NAS when compared to methadone, the risk of adverse obstetrical outcomes was similar in both buprenorphine and methadone-treated women [10–12]. Previously conducted studies observed an association between prenatal cannabis exposure and adverse neonatal outcomes, including preterm birth, reduced birth weight, small for gestational age, and admission to the neonatal intensive care unit [13–15]. Nevertheless, this association was largely confounded by concomitant tobacco use [13–15].

Numerous studies have been conducted in the past decades to assess the effect of prenatal cocaine and opioid use on maternal and neonatal outcomes. However, many of these studies used reference groups, including women who did not use substances [5, 16–18]. Such reference groups could often vary significantly from the substance-using exposed groups in terms of risk factors (like medical care provided to them, socioeconomic status, and smoking status in pregnancy) associated with polysubstance use in pregnancy [5, 16–18]. Using such reference groups that do not use any substances limits the ability of the study to distinguish the effect of risk factors associated with polysubstance use in pregnancy from the effects of polysubstance use in pregnancy itself [19].

To address this gap, we conducted an observational study on a population of pregnant women, aiming to compare the risk of adverse obstetrical and neonatal outcomes between those who consumed cocaine and opioids during their pregnancy and those who only consumed cannabis during pregnancy. All participants were followed in the same perinatal addiction consultation.

## Methods

### Study design

This is an observational study conducted using data collected between 2005 and 2014 from pregnant women followed for current or past addiction at the specialized consultation (Addi-Vie) at the CHUV maternity hospital in Lausanne, Switzerland. The ethical committee of Canton de Vaud approved the study (Protocol number: 76/15). The data for the study was collected during the follow-up of the pregnant women from the beginning of their pregnancy until the birth of the child. The follow-up at the addiction consultation at the CHUV maternity hospital was conducted by midwives under the medical supervision of the obstetricians.

## Inclusion and exclusion criteria

Pregnant women followed at the perinatal addiction consultation and reporting active use of cannabis, cocaine, or opioids (illicit use or under opioid agonist therapy) such as heroin, methadone, and buprenorphine were included in the study. Women who did not report active consumption of the drugs mentioned above were excluded from the study. Twin pregnancies were also excluded as they increased the risk for preterm birth, lower birth weight, and lower Apgar score, i.e., Apgar score less than 7 at 5 min [20, 21].

## Study groups

The exposed group included pregnant women reporting active consumption of either cocaine, opioids (including illicit use of heroin or those under opioid agonist therapy like methadone and buprenorphine), or both, with or without cannabis at the beginning of the pregnancy. Pregnant women reporting the use of cannabis alone at the beginning of the pregnancy were included in the reference group.

## Baseline characteristics and potential confounders

Information on several socio-demographic and maternal characteristics was collected at baseline from the pregnant women in the study cohort. Socio-demographic information included maternal age (women older than 35 years), nationality (Swiss or other nationals), relationship status (single—yes/no), experience of domestic violence (physical violence, associated or not with verbal violence), availability of support from friends or family during pregnancy (good support/poor or no support), employment status (employed/unemployed/ with disability insurance), and accommodation (yes/no). We also collected information on maternal characteristics, including nulliparity, unplanned pregnancy, psychological conditions (anxiety or depression, personality disorder, bipolar disorder, psychotic disorder, and eating disorder), prescribed psychoactive treatment (benzodiazepines, antipsychotics, antidepressants, hypnotics, and other), viral infections such as HIV, hepatitis B (Hep B) (chronic/active), and hepatitis C (Hep C) (chronic/active), and other pre-pregnancy medical comorbidities that could potentially affect the prognosis of the pregnancy (e.g., epilepsy, hypertension etc.,). Additionally, baseline information included details on tobacco and alcohol use during pregnancy, history of illicit drug use, and any medical care for addiction or opioid agonist therapy prior to pregnancy.

## Outcomes

The primary outcomes of the study included two composite adverse outcomes: i. adverse obstetrical composite outcome, and ii. adverse neonatal composite outcome. The adverse obstetrical composite outcome consisted of the presence of at least one of the following outcomes: preterm birth (delivery occurring at less than 37 weeks of gestation), induction of labor (for maternal and fetal pathological reasons such as intrauterine growth restriction, premature rupture of membrane, pre-eclampsia, cholestasis, diabetes, decrease in fetal movements, abnormal uterine and umbilical dopplers, and exceeding term), instrumental birth (delivery of baby using forceps or the vacuum cup due to non-progression of the fetus or due to fetal distress), and emergency cesarean-section. The adverse neonatal composite outcome included the presence of at least one of the following outcomes: SGA (birth weight below the 10th percentile for gestational age [22]), diagnosis of NAS (diagnosed by pediatricians and symptoms include hyperirritability, high-pitched crying, sneezing, and diarrhea [23]), Apgar score less than 7 at 5 min, respiratory distress syndrome (breathing disorder occurring after birth due to deficiency of surfactant in the lungs of the newborn [24]), congenital malformations (identified at first pediatric consultation)**,** neonatal infection (infections in neonatal period potentially caused by premature rupture of membrane or nosocomial infections or due to suspected chorioamnionitis), and hospitalization for more than 5 days (in the neonatal department/children’s hospital in CHUV, Lausanne).

The frequency and proportion of individual adverse obstetrical and neonatal outcomes included in the two composite outcomes (adverse obstetrical and neonatal composite outcomes) were reported as the secondary outcomes of this analysis.

## Statistical analysis

Descriptive analyses were conducted to summarize the baseline characteristics and individual components of adverse obstetrical and neonatal composite outcomes. Frequency and proportion were reported for categorical variables. For continuous variables that were normally distributed, mean and standard deviation (SD) were reported, and for those with skewed distribution, median and interquartile range (IQR) were reported. The association between maternal cocaine and/or opioid exposure during pregnancy and i. adverse obstetrical composite outcomes, and ii. adverse neonatal composite outcomes were assessed using multivariable logistic regression. Based on literature evidence and expert advice (AP and EM), directed acyclic graphs (DAGs) (See Supplementary Figures S1 and S2, Additional File 1) were constructed using baseline characteristics that were associated with the exposure and were known risk factors of outcome to identify the potential confounders for statistical analysis. This includes maternal age [25], nationality [26], single status [27], unplanned pregnancy [28], alcohol use [29], history of drug use / medical care for addiction/ Opioid agonist therapy prior to pregnancy [30–32], psychological condition or psychoactive medication [33–37], HIV, hepatitis B or C infection [38–44], other medical comorbidities prior to pregnancy which could potentially affect the prognosis of the pregnancy [45–47], domestic violence [48], poor support from family and friends [49–51], and disability insurance [52]. For the analysis of the neonatal composite outcome, the model was also adjusted for the sex of the child. Adjusted Odds Ratio (AOR) and 95% Confidence Interval (CI) were calculated by controlling for these potential confounders in the logistic regression analysis. Following the regression modeling to control for confounding, Inverse Probability of Treatment Weighting (IPTW) was conducted. For each pregnant woman included in this study, we calculated the probability of having an adverse outcome using a logistic regression model with exposure status as the dependent variable and all the potential confounders as independent variables. Weights were created for individual women in the exposed group by calculating the inverse of these predicted values and the inverse of the 1-predicted value for patients in the reference group. The weights were stabilized and were truncated at 5 (99th percentile) to avoid extreme weights. The balance of covariates was assessed using the standardized mean differences (SMD), and the full balance was achieved when the SMD values were between 0.1–0.25. Once an acceptable balance of covariates was achieved, conditional odds ratio (OR_conditional_) and 95%CI were estimated using weighted logistic regression.

Listwise deletion was performed to include only pregnant women with complete data for all potentially confounding covariates, both for the regression and IPTW analysis.

We conducted post-hoc sensitivity analyses to test for composite outcome fallacy by assessing the association between prenatal cocaine and/or opioid exposure and i. modified adverse obstetrical composite outcome excluding “induced labor” (defined as the presence of any/all of the following: preterm birth, instrumental birth, and emergency c-section) and ii. modified adverse neonatal composite outcome excluding “NAS” (defined as the presence of any/all of the following outcomes: SGA, Apgar score less than 7, respiratory distress syndrome, congenital malformations, neonatal infections, and hospitalization for more than 5 days), using a statistical model similar to our primary analysis. All the statistical analysis for this study was conducted using R version 4.2.2.

## Subgroup analysis

We conducted a subgroup analysis where we assessed the subgroups of patients with and without psychoactive treatment during pregnancy to understand the impact of psychoactive drugs on premature birth & other adverse neonatal outcomes. A descriptive analysis was performed, and frequency, along with the prevalence of these adverse outcomes, was reported.

## Results

**Fig. 1:**
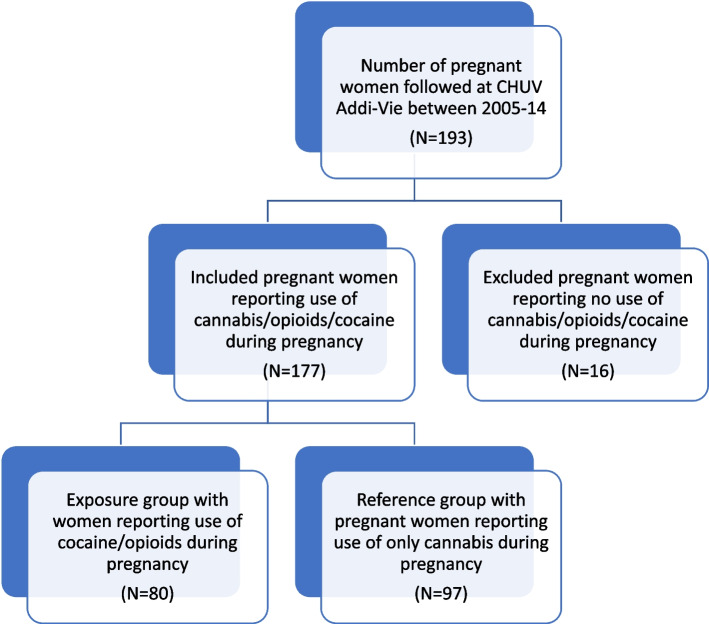
Flowchart of Study groups

From 2005 to 2014, 193 women were followed up at CHUV-Addi-Vie consultation for their addiction problem during pregnancy. We included 177 out of 193 women who met the inclusion criteria for the study. The exposed group consisted of 80 pregnant women, while the reference group included 97 pregnant women (Fig. 1).

## Baseline characteristics

The baseline maternal characteristics have been summarized and displayed in Table 1. In our study group, women in the exposed group were comparatively older (31.6 years vs 26.9 years). Most of the pregnant women in the exposed group had a history of drug use or medical care for addiction treatment or opioid agonist therapy prior to pregnancy (83.8% vs 41.2%). Many pregnant women in the exposed group were also diagnosed with a psychiatric condition or were prescribed a psychoactive treatment during pregnancy (57.5% vs 21.6%). Additionally, a higher proportion of pregnant women in the exposed group were Swiss nationals (76.3% vs. 66.0%), had unplanned pregnancies (63.8% vs. 54.6%), consumed alcohol (16.3% vs. 8.2%), had HIV, Hep B, or Hep C infection (47.5% vs. 14.4%), reported domestic violence (22.5% vs. 17.5%), had poor or no support from friends and family during pregnancy (28.8% vs. 17.5%) and had disability insurance (15.0% vs. 4.1%).

**Table 1:** Baseline characteristics

	**Exposed group**	**Reference group**	**SMD**
	**(*N* = 80)**	**(*N* = 97)**	
**DEMOGRAPHIC CHARACTERISTICS**			
** Age *mean(sd)***	31.6(± 5.9)	26.9(± 5.6)	
Women of age > 35 years	26 (32.5%)	5(5.2%)	0.747
**Nationality *N (%)***			0.228
Swiss	61(76.3%)	64(66.0%)	
Other nationals	19(23.8%)	33(34.0%)	
**Single *N (%)***			0.036
Yes	10(12.5%)	11(11.3%)	
No	70(87.5%)	86(88.7%)	
**MATERNAL CHARACTERISTICS**			
**Tobacco and alcohol use *N (%)***			
Tobacco use	73 (91.3%)	80(82.5%)	0.262
Alcohol use	13(16.3%)	8 (8.2%)	0.246
**Nulliparous pregnancy *N (%)***	46 (57.5%)	61 (62.9%)	0.110
**Unplanned pregnancy *N (%)***^***#***^	51 (63.8%)	53(54.6%)	0.192
**Missing**	1 (1.3%)	1(1.0%)	
**History of drug use/medical care/ opioid agonist therapy before pregnancy *N(%)***	67(83.8%)	40 (41.2%)	0.977
Drug use	53(66.3%)	39 (40.2%)	
Medical care for addiction	57 (71.3%)	5 (5.2%)	
Opioid agonist therapy	52 (65.0%)	2(2.1%)	
**Psychiatric conditions or Psychoactive treatment *N(%)***^***#***^	46(57.5%)	21(21.6%)	0.788
**Number of diagnosed psychiatric conditions**			
0	48(60.0%)	76(78.4%)	
1	26(32.5%)	19(19.6%)	
2	6(7.5%)	2(2.1%)	
** Missing**	1(1.3%)	0	
**Types of psychiatric conditions diagnosed**			
Anxiety and depression	15(18.8%)	8 (8.2%)	
Personality disorders	15(18.8%)	7(7.2%)	
Bipolar disorder	3(3.8%)	0	
Psychotic disorders	3(3.8%)	5 (5.2%)	
Eating disorder	2(2.5%)	3 (3.1%)	
**Types of prescribed psychoactive treatment**	37(46.2%)	10(10.3%)	
Benzodiazepines	31 (38.8%)	5 (5.2%)	
Antipsychotics	7 (8.8%)	5 (5.2%)	
Hypnotics	11 (13.8%)	2 (2.1%)	
Anti-depressants	13 (16.3%)	5 (5.2%)	
Other	0	1(1.0%)*	
**Number of prescribed psychoactive treatment**			
0	43(53.8%)	87(89.7%)	
1	18(22.5%)	4(4.1%)	
2	14(17.5%)	4(4.1%)	
3	4(5.0%)	2(2.1%)	
4	1(1.3%)	0	
**Active or chronic viral infections (HIV, Hep B and Hep C) *N(%)***	38 (47.5%)	14 (14.4%)	0.766
**Number of active/chronic viral infection**			
0	42(52.5%)	83(85.6%)	
1	23(28.8%)	13(13.4%)	
2	11(13.8%)	1(1.0%)	
3	4(5.0%)	0	
**Types of active/chronic viral infections**			
Active HIV	5(6.3%)	1 (1.0%)	
Active Hepatitis B	2(2.5%)	0	
Active Hepatitis C	30(37.5%)	5 (5.2%)	
Chronic Hepatitis B	13(16.3%)	6(6.2%)	
Chronic Hepatitis C	7(8.8%)	3(3.1%)	
** Other medical comorbidities before pregnancy *N(%)***	19 (23.8%)	28 (28.9%)	0.116
**SOCIAL AND LIFESTYLE FACTORS**			
**Domestic violence *N(%)***			0.125
Verbal and physical violence	18 (22.5%)	17 (17.5%)	
Physical violence	4 (5.0%)	9 (9.3%)	
**Support from family/friends during pregnancy *N(%)***			0.269
Good support	57 (71.3%)	80 (82.5%)	
Poor/no support	23(28.8%)	17(17.5%)	
**Job and source of income *N(%)***			0.527
Employed	10 (12.5%)	33(34.0%)	
Disability insurance	12 (15.0%)	4(4.1%)	0.376
**Accommodation *N(%)***			0.226
Yes	78 (97.5%)	97(100%)	
No	2(2.5%)	0	

Note: Few (1.13%, *N* = 2) missing information was observed for variables on unplanned pregnancy and diagnosed psychiatric conditions. These women were removed through list-wise deletion before conducting regression analysis

^*^Consumed morphine

*SMD* Standardized mean differences

## Cocaine and/or opioid exposure

**Fig. 2:**
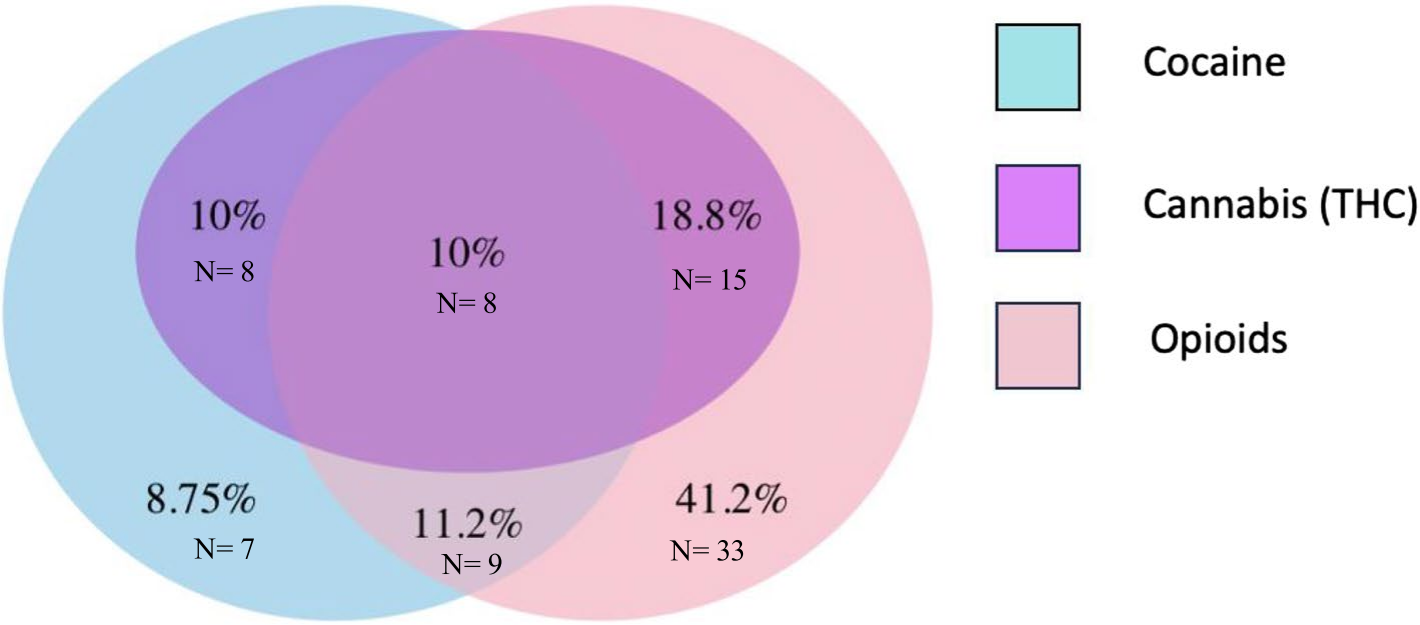
The Venn diagram describes the drug use in the exposure group: cocaine(39.9%), cannabis(38.8%), and opioids (81.2%)

In the exposed group, 81.2% (*n* = 65) of women reported opioid consumption, and 39.9% (*n* = 32) were exposed to cocaine (Fig. 2). Pregnant women who consumed only cocaine constituted 8.8% (*n* = 7) of the exposed group. Concurrent use of cocaine and opioids was observed in 21.2% (*n* = 17) of women. Among opioid users, 41.2% (*n* = 33) of pregnant women reported consumption of only opioids (heroin, methadone, or buprenorphine). The most used opioid among pregnant women in the exposed group was methadone, reported by 72.5% (*n* = 58) of women, followed by heroin 31.3% (*n* = 25), and buprenorphine 6.3% (*n* = 5). Although 31.25% (*n* = 25) of opioid users consumed heroin, only three women used heroin alone, while 88.0% (*n* = 22) of them concurrently used methadone. Only 1 woman (1.3%) consumed heroin with methadone and buprenorphine. Figure 2 describes the overlapping of drug use in the exposed group.

## Adverse obstetrical and neonatal composite outcomes

Overall, 108 (61.0%) mothers reported experiencing at least one or all components in the adverse obstetrical composite outcome. This includes 44 (55.0%) mothers from the exposed group and 64 (65.9%) mothers from the reference group. The crude odds ratio (OR_crude_) of adverse obstetrical composite outcome in the exposed group compared to the reference group was OR_crude_ 0.63 (95% CI: 0.34–1.15). After adjusting for potential confounders in a multivariable logistic regression model, prenatal cocaine and/or opioid exposure was associated with reduced odds of adverse obstetrical composite outcome (AOR 0.37, 95% CI: 0.16–0.87). Controlling for confounders using IPTW also yielded a similar OR_conditional_ of 0.39 (95% CI: 0.17–0.88) for the adverse obstetrical composite outcomes (Table 2).

**Table 2:** Association between the adverse obstetrical and neonatal composite outcomes and cocaine/opioid exposure during pregnancy

Composite outcomes	Total observations (*N* = 177)	Exposed group (*N* = 80)	Reference group (*N* = 97)	Crude Odds ratio (95%CI)	Adjusted Odds ratio^1^(95%CI)	*p*-value^1^	Conditional Odds ratio^2^ (95%CI)	*p*-value^2^
Adverse obstetrical composite outcome^a^	108 (61.0%)	44(55.0%)	64(65.9%)	0.63(0.34–1.15)	0.37(0.16–0.87)	0.023*	0.39(0.17–0.88)	0.026*
Adverse neonatal composite outcome^b^	141 (79.7%)	72 (90.0%)	69 (71.1%)	3.65(1.56–8.56)	2.79(0.95–8.23)	0.063	3.88(1.23–12.23)	0.022*

^1^The Adjusted Odds ratio was obtained through the multivariable logistic regression i.e., logit(y) = β_0 _+ β_1_ x_1_ + .. + β_n_x_n_ where y is the outcomes and (x_1_,x_2_,..x_n_) represent the exposure and other covariates

^2^The Conditional Odds ratio was obtained through inverse probability treatment weighting (IPTW). The propensity score for IPTW was derived through a logistic regression model. SMD value for the model was between 0.1–0.25, except for variables on psychiatric conditions and psychoactive medication and poor/no support

^a^The model was adjusted for the exposure and covariates such as maternal age, nationality, relationship status, alcohol use, history of drug use/opioid agonist therapy/medical care for addiction, active or chronic viral infections, unplanned pregnancy, other medical comorbidities, domestic violence, poor/no support, disability insurance

^b^The model was adjusted for the exposure and covariates such as maternal age, nationality, sex of the child, relationship status, alcohol use, history of drug use/opioid agonist therapy/medical care for addiction, active or chronic viral infections, unplanned pregnancy, other medical comorbidities, domestic violence, poor/no support, disability insurance

Exposed group includes pregnant women reporting use of cocaine and/or opioids, along with or without cannabis during pregnancy and reference group includes women who reported use of only cannabis during pregnancy. In this analysis, 175 women were included (2 removed by list-wise deletion). The exposed group 79 women and the reference group included 96 women in this analysis

^*^p < 0.05; VIF values of all the independent variables (IV) in the model is less than 5, thus no evidence of multicollinearity

In our study, 141 (79.7%) infants were observed to experience at least one or all components of the adverse neonatal composite outcome. While 72 (90.0%) infants experiencing such outcomes reported prenatal cocaine and/or opioid exposure, 69 (71.1%) infants reported prenatal cannabis exposure. The OR_crude_ estimate for the adverse neonatal composite outcome was 3.65 (95%CI: 1.56–8.56) in women included in the exposed group compared to the reference group. After adjusting for several demographic, maternal, and lifestyle factors, the AOR was 2.79 (95%CI: 0.95–8.23). After using IPTW strategy to adjust for the confounders, the OR_conditional_ was 3.88 (95% CI: 1.23–12.23) (Table 2).

## Individual adverse obstetrical and neonatal composite outcomes

Table 3 summarizes the individual adverse obstetrical events observed in the exposed and reference groups. More women in the exposed group reported preterm birth (17.5% vs 13.4%).

**Table 3:** Descriptive summary of individual adverse obstetrical outcomes

Individual adverse obstetrical outcomes	Total observations (*N* = 177)	Exposed group (*N* = 80)	Reference group (*N* = 97)
**Premature birth N (%)**	27 (15.3%)	14 (17.5%)	13(13.4%)
34–37 weeks	17(62.9%)	7 (50.0%)	10 (76.9%)
Less than 34 weeks	10(37.0%)	7 (50.0%)	3 (23.1%)
**Induced labor N (%)**	65(36.7%)	23(28.8%)	42(43.3%)
**Instrumental delivery N (%)**	13(7.3%)	7(8.8)	6(6.2%)
With forceps	11(84.6%)	6 (85.7%)	5(83.3%)
With vacuum	2(15.4%)	1(14.3%)	1(16.7%)
**Emergency caesarean section N (%)**	37(48.7%)	18 (40.0%)	19 (61.3%)

Individual adverse neonatal outcomes are presented in Table 4. Higher number of Infants from the exposed group were SGA (35.0% vs 25.8%), experienced respiratory distress syndrome (21.3% vs 11.3%), and were hospitalized for more than 5 days (65.0% vs 10.3%). While no infant in the reference group was diagnosed with NAS, more than half of the infants in the exposed group were diagnosed with NAS (57.5% vs 0%).

**Table 4:** Descriptive summary of individual adverse neonatal outcomes

Individual adverse neonatal outcomes	Total observations (*N* = 177)	Exposed group (*N* = 80)	Reference group (*N* = 97)
Small for gestational age *N (%)*	53(29.9%)	28(35.0%)	25(25.8%)
Neonatal withdrawal syndrome *N (%)*	46(25.9%)	46(57.5%)	0
Apgar score less than 7 *N (%)*	4(2.3%)	1(1.3%)	3(3.1%)
Respiratory distress syndrome *N (%)*	28(15.8%)	17(21.3%)	11(11.3%)
Congenital malformations *N (%)*	4(2.3%)	1(1.3%)	3(3.1%)
Neonatal infection *N (%)*	5(2.8%)	3(3.8%)	2(2.1%)
Hospitalization for > 5 days *N (%)*	62(35.0%)	52 (65.0%)	10 (10.3%)

## Subgroup analysis

The results of the subgroup analysis are described in Table 5. In the exposed group, a higher proportion of infants born to women receiving psychoactive treatment were SGA (40.5% vs 30.2%) and had respiratory distress syndrome (35.1% vs 9.3%). Within the exposed group, women receiving psychoactive treatment reported a higher number of preterm births (29.7% vs 7.0%), with a larger number delivering infants before 34 weeks of gestation (16.2% vs 2.3%). Most infants from the exposed group receiving psychoactive treatment were diagnosed with NAS (89.2% vs 30.2%). A vast proportion of the newborns from the exposed group receiving psychoactive medication in pregnancy were also hospitalized for more than 5 days (97.3% vs 37.2%).

**Table 5:** Preterm births and individual adverse neonatal outcomes in subgroups receiving and not receiving psychoactive medication

Individual adverse neonatal outcomes	Total observations (*N* = 177)		Exposed group		Reference group	
	**With psychoactive medication (*N* = 47)**	**Without Psychoactive medication (*N* = 130)**	**With psychoactive medication (*N* = 37)**	**Without Psychoactive medication (*N* = 43)**	**With psychoactive medication (*N* = 10)**	**Without Psychoactive medication (*N* = 87)**
**Premature birth *N* (%)**	11(23.4%)	16 (12.3%)	11(29.7%)	3(7.0%)	0	13(14.9%)
Between 34–37 weeks	5 (10.6%)	12 (9.2%)	5(13.5%)	2(4.7%)	0	10(11.5%)
Less than 34 weeks	6 (12.8%)	4 (3.1%)	6(16.2%)	1(2.3%)	0	3(3.4%)
**Small for gestational age *N (%)***	20 (42.6%)	33(25.4%)	15(40.5%)	13(30.2%)	5(50.0%)	20(23.0%)
**Neonatal withdrawal syndrome *N (%)***	33 (70.2%)	13(10.0%)	33(89.2%)	13(30.2%)	0	0
**Apgar score less than 7 *N (%)***	1 (2.1%)	3(2.3%)	0	1(2.3%)	1(10.0%)	2(2.3%)
**Respiratory distress syndrome *N (%)***	15 (31.9%)	13(10.0%)	13(35.1%)	4(9.3%)	2(20.0%)	9(10.3%)
**Malformations *N (%)***	1 (2.1%)	3(2.3%)	1(2.7%)	0	0	3(3.4%)
**Neonatal infections *N (%)***	1 (2.1%)	4(3.1%)	1(2.7%)	2(4.7%)	0	2(2.3%)
**Hospitalization for > 5 days *N (%)***	38 (80.9%)	24(18.5%)	36(97.3%)	16(37.2%)	2(20.0%)	8(9.2%)

### Results of post-hoc sensitivity analysis

On conducting the post-hoc sensitivity analysis, no association was observed between prenatal cocaine and/or opioid exposure and modified adverse obstetrical composite outcome, excluding induced labor (OR_crude_: 1.16(0.63–2.16), AOR: 0.70(0.31–1.59), p = 0.394, OR_conditional_: 0.84(0.37–1.92), p = 0.681). On the contrary, in analysis with modified adverse neonatal composite outcome excluding NAS, the crude analysis showed an association between the prenatal cocaine and/or opioid exposure and the modified adverse neonatal composite outcome (OR_crude_: 3.20, 95%CI: 1.41–7.28, p = 0.005*). However, this association was no more significant after controlling for confounders in a multivariable logistic regression analysis (AOR: 2.46, 95% CI: 0.85–7.12, p = 0.109). Nevertheless, on conducting the weighted logistic regression analysis, there was still a significant association between prenatal cocaine and/or opioid exposure and modified adverse neonatal composite outcome excluding NAS (OR_conditional_: 3.52, 95% CI: 1.16–10.71, p = 0.037*). The results of the post-hoc sensitivity analyses are presented in Table 6.

**Table 6:** Results of post-hoc sensitivity analyses

Composite outcomes	Total observations (*N* = 177)	Exposed group (*N* = 80)	Reference group (*N* = 97)	Crude Odds ratio (95%CI)	Adjusted Odds ratio^1^(95%CI)	*p*-value^1^	Conditional Odds ratio^2^ (95%CI)	*p*-value^2^
Adverse obstetrical composite outcome^a^	63 (35.6%)	30(37.5%)	33(34.0%)	1.16 (0.63–2.16)	0.70(0.31–1.59)	0.394	0.84(0.37–1.92)	0.681
Adverse Neonatal composite outcome^b^	140(79.1%)	71(88.8%)	69(71.1%)	3.20(1.41–7.28)	2.46 (0.85–7.12)	0.109	3.52 (1.16–10.71)	0.037*

^1^The Adjusted Odds ratio was obtained through the multivariable logistic regression i.e., logit(y) = β_0_ + β_1_x_1_ + … + β_n_x_n_ where y is the outcomes and (x_1_,x_2_,..x_n_) represents the exposure and other covariates

^2^The Conditional Odds ratio was obtained through inverse probability treatment weighting (IPTW). The propensity score for IPTW was derived through a logistic regression model. SMD value for the model was between 0.1–0.25, except for variables on psychiatric conditions and psychoactive medication and poor/no support

^a^The model was adjusted for the exposure and covariates such as maternal age, nationality, relationship status, alcohol use, history of drug use/opioid agonist therapy/medical care for addiction, active or chronic viral infections, unplanned pregnancy, other medical comorbidities, domestic violence, poor/no support, disability insurance. ^b^The model was adjusted for the exposure and covariates such as maternal age, nationality, sex of the child, relationship status, alcohol use, history of drug use/opioid agonist therapy/medical care for addiction, active or chronic viral infections, unplanned pregnancy, other medical comorbidities, domestic violence, poor/no support, disability insurance

In the above model, the adverse obstetrical composite outcome is defined as the presence of any or all of the following adverse outcomes: preterm birth (birth occurring less than 37 weeks of gestation), Instrumental birth (through forceps or suction, in cases of fetal distress or non-progression of fetal presentation), and emergency c-section. The adverse neonatal composite outcomes are defined as the presence of any or all of the following adverse outcomes: SGA, Apgar score less than 7, respiratory distress syndrome, congenital malformations, neonatal infections, and hospitalization for more than 5 days

The exposed group includes pregnant women reporting use of cocaine and/or opioids, along with or without cannabis during pregnancy and the reference group includes women who reported use of only cannabis during pregnancy. In this analysis, 175 women were included (2 removed by list-wise deletion). The exposed group was 79 women, and the reference group included 96 women in this analysis

^*^p < 0.05; VIF values of all the independent variables (IV) in the model is less than 5, thus no evidence of multicollinearity

## Discussion

We conducted an observational study to compare the risk of adverse obstetrical and neonatal composite outcomes in women reporting the use of cocaine and/or opioids during pregnancy and women reporting the use of only cannabis during pregnancy. We observed a significant association between prenatal exposure to cocaine and opioids and the adverse neonatal composite outcome. The odds of adverse neonatal composite outcome in pregnant women reporting use of cocaine and opioids during pregnancy was 3.88 times higher than the odds of adverse neonatal composite outcome in pregnant women reporting use of only cannabis during pregnancy. Additionally, prenatal use of cocaine and opioids was also associated with reduced odds of adverse obstetrical composite outcomes when compared to prenatal cannabis use in our study. In absolute numbers, many infants in the exposed group reported SGA, NAS, respiratory distress syndrome, hospitalization for more than 5 days, and preterm birth.

Available literature evidence does illustrate the effect of opioids and cocaine in inducing adverse pregnancy outcomes. A meta-analysis of 80 observational studies on the impact of prenatal opioid exposure on neonatal outcomes found increased odds of preterm birth, reduction in birth weight, and prolonged hospitalization with in-utero opioid exposure [18]. Quantitative analysis assessing the association between the use of cocaine by pregnant women and adverse neonatal outcomes also concluded that in-utero cocaine exposure increased the risk of preterm birth and SGA [7]. The exact mechanism of action through which cocaine and opioids drive these adverse outcomes is unknown. It is speculated that cocaine use in pregnancy might activate the adrenergic systems, which could induce vasoconstriction, hypertension, and increased catecholamines in the user, thus driving the observed adverse obstetrical and neonatal outcomes [16]. On the other hand, opioids cross the placenta in pregnancy and potentially impact the development and functioning of the placenta [53–55]. This could lead to preterm birth and restricted growth of the fetus [53–55].

We observed NAS in more than half of the infants in our exposed group. This observation could potentially be attributed to the high prevalence (81.4%) of opioid consumption among pregnant women in our exposed group, with 89.2% of them consuming methadone during pregnancy. NAS is often associated with opioid consumption during pregnancy [56]. Although the pathophysiology of NAS is still unclear, the abrupt halt of opioids post-birth results in the central, peripheral, and autonomic nervous systems manifestations characteristic of NAS [54, 56, 57]. Among opioids, consumption of methadone has been associated with a higher incidence of NAS compared to buprenorphine and heroin, although the presence of a dose-dependent relationship between methadone exposure and NAS has not been elucidated [58, 59]. Research to identify the effects of concurrent use of opioids with cocaine has resulted in contradicting observations, with few studies observing increased frequency of NAS during concurrent use, while others claim the attenuation of NAS with the use of cocaine and opioids [60–62]. Therefore, further investigation is required to assess the impact of the concurrent use of cocaine and opioids on NAS [61]. In our study cohort, NAS was solely observed in the exposed group. Thus, post-hoc sensitivity analysis was conducted to test the composite outcome fallacy. The results of this analysis underscored that prenatal cocaine and/or opioid exposure also increases the odds of other adverse neonatal outcomes apart from NAS.

In this study, we also observed a significant association between prenatal cocaine and/or opioid exposure and reduced odds of adverse obstetrical composite outcomes when compared to in-utero cannabis exposure. However, this association turned insignificant following the exclusion of induced labor from the adverse obstetrical composite outcome in the post-hoc sensitivity analysis. This suggests that the association observed between prenatal cocaine and/or opioid exposure and adverse obstetrical composite outcome in the primary analysis could potentially be driven by induced labor, that is per se a composite outcome as it is the consequence of various obstetrical and neonatal conditions. More studies with large sample sizes are needed to disentangle the importance of these conditions.

Infants exposed to psychoactive medications such as benzodiazepines, antidepressants, antipsychotics, and hypnotics showed a higher incidence of adverse outcomes, including preterm birth, SGA, NAS, and hospitalization for more than 5 days. This observation is in line with previously conducted meta-analyses which have shown that use of benzodiazepines, antidepressants, and antipsychotics in pregnant women can increase the risk of preterm birth, SGA, respiratory distress syndrome, withdrawal, and hospitalization in the neonatal intensive care unit [35, 37, 63–66]. In our study, the psychoactive treatment potentially contributed to the adverse neonatal events acting synergistically with the consumed cocaine and/or opioids during pregnancy [67]. A population-based retrospective cohort study conducted in Tennessee observed one such synergistic effect when infants with in-utero exposure to benzodiazepines along with opioid exposure had more than 50% increased odds of developing a neonatal abstinence syndrome that required pharmacological intervention [68]. However, there is a paucity of evidence assessing a possible synergistic effect of psychoactive treatment when taken along with substances such as cocaine and/or opioids in causing adverse neonatal and obstetrical outcomes. Psychological illness is a risk factor for substance abuse [6]. Pregnant women frequently experience mood and anxiety disorders, which when left untreated, results in psychiatric episodes, relapse, poor nutrition intake, substance use, and adverse neonatal outcomes [69–71]. Thus, it is important for future research to focus on the potential additive effect of psychoactive drugs when taken concomitantly with other drugs like cocaine and opioids during pregnancy.

## Strengths and limitations

The current observational study has numerous strengths. For this study, comprehensive information on several social and demographic details of the participating subjects was extracted. This, in turn, allowed us to account for various confounders that otherwise affect the observed association between exposure and adverse obstetrical and neonatal outcomes. Observational studies conducted earlier found it challenging to identify a comparable reference group for assessing the impact of substance use during pregnancy on perinatal outcomes [18]. The choice was limited to the unexposed reference group (i.e. reference group who do not use any illicit substances) visiting the same healthcare setting as the pregnant substance users. However, this tends to induce confounding due to differences in several sociodemographic characteristics between substance users and non-users. To control for such confounding, previous studies did not often use matching or other statistical adjustment techniques [18]. In our study, for better control of confounders, we selected patients consuming only cannabis during pregnancy as our reference group. Furthermore, pregnant women in both the reference and exposed groups came from the same perinatal consultation, where they were potentially ascertained to be followed up similarly from the beginning of the pregnancy until childbirth. This shared context increased the comparability of the groups not limited to lifestyle and other characteristics, such as alcohol consumption, but also in terms of counseling and medical care. This approach allowed for a more comprehensive consideration of unmeasured or unknown factors associated with substance use, a distinction that may not have been as effectively addressed when using reference groups who do not use illicit substances or healthy controls. Additionally, the confounders associated with illicit drug use were effectively accounted for by using the IPTW method.

**Fig. 3:**
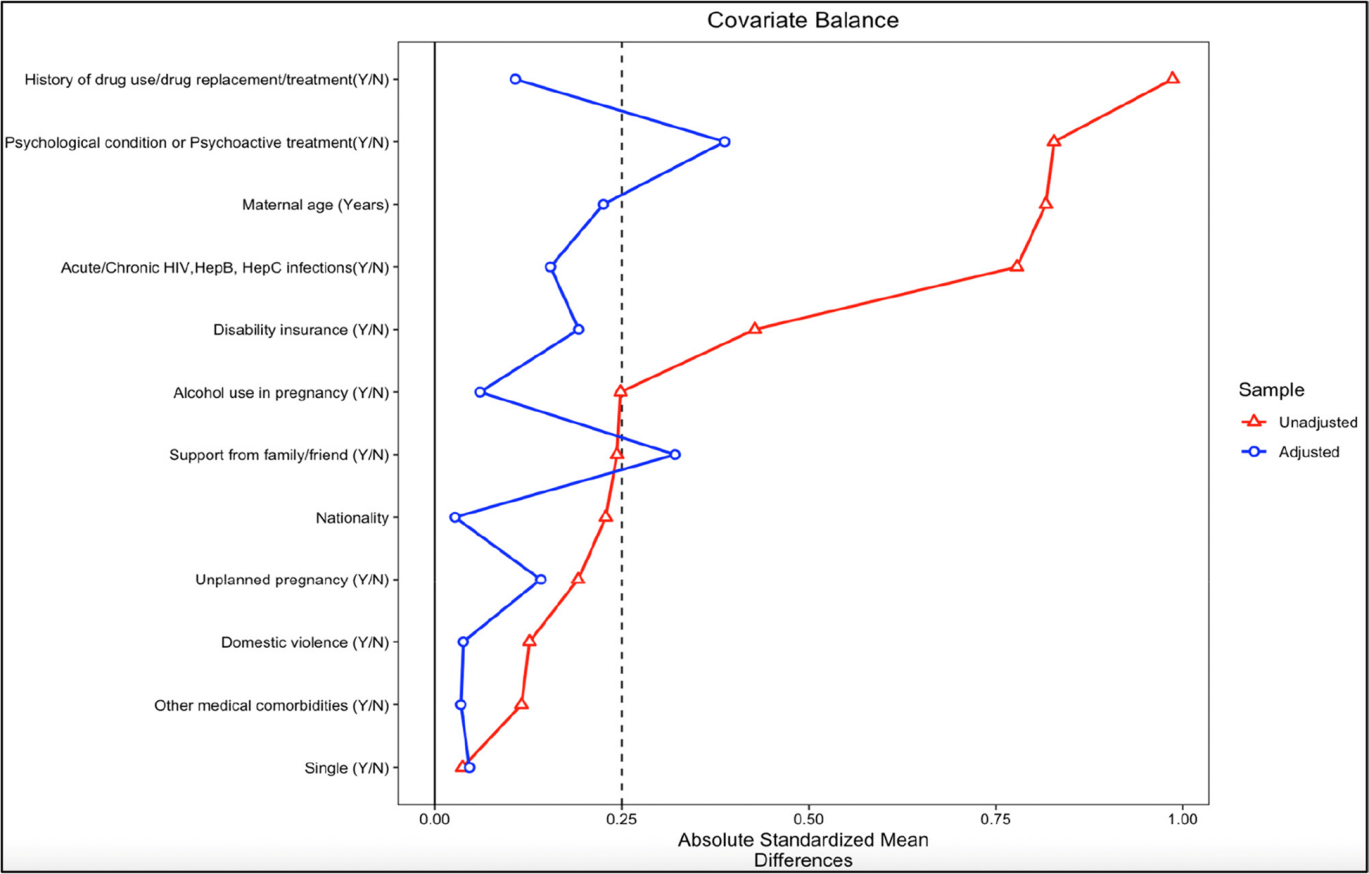
Covariate balance plot for IPTW: overall balance of covariates improved but variables depicting “psychological conditions or psychoactive conditions and support from family/friend” were not completely balanced (not within the threshold of 0.1- 0.25)

Besides, it is also important to note that this study had some limitations. Firstly, women in our exposed group also used cannabis. This made it difficult to isolate the effects of cocaine and opioids specifically and lowered the effect size observed. However, this reflects the real-world substance use patterns, where polysubstance use is highly prevalent. Indeed, cannabis is often used with other substances, including cocaine and opioids, and excluding those using cannabis from the exposed group may decrease the ecological validity of our study. Secondly, though the use of cocaine, opioids, and cannabis was reported at the beginning of pregnancy, information on the gestational age at which the substance use was reported, frequency, amount, duration of substance use, and duration of opioid agonist therapy during pregnancy was not collected. Additionally, although all the women were treated for their current or past addiction at CHUV maternity hospital, the specific details of the treatment provided to the women in our study cohort were not available. Third, while analyzing the association between prenatal exposure to cocaine and/or opioids and adverse obstetrical and neonatal composite outcomes using IPTW, the overall balance of individual covariates improved greatly. However, complete balance was not achieved (i.e., the SMD value was not between 0.1–0.25 for all covariates) on covariates describing presence of psychological conditions or psychoactive medications and poor support from family/friends (Fig. 3). Fourth, despite only a few individuals reporting to have used alcohol (13 in the exposed and 8 in the reference group) in our study, the model was still adjusted for alcohol use.

Another limitation of the study is the small sample size, making it difficult to obtain accurate estimates while assessing for the association between cocaine and/or opioid exposure in pregnancy and adverse obstetrical and neonatal composite outcomes. It also limited us from disentangling the effect of the psychoactive medications and prenatal cocaine and/or opioid exposure when used concurrently, on preterm birth and other adverse neonatal outcomes. Finally, the self-reported drug use in this study also raises a concern of underreporting [72]. Legal consequences like child apprehension could discourage women from self-reporting substance use [73]. However, the mother–child dyads treated in this study at the CHUV-Addivie addiction clinic were protected whenever possible, and about 70% of the mothers treated at this addiction clinic returned home with their infants. Thus, the risk of bias due to exposure misclassification arising from underreporting due to fear of child apprehension is minimal in this setting [74]*.*

Additionally, the stigma associated with substance use might also result in underreporting as it could prevent substance users from accessing antenatal care at the early stages of pregnancy [75]. A community-based approach involving interprofessional healthcare networks with clinicians, nurses, midwives, community pharmacists, and social workers can play an important role in improving access to antenatal care for pregnant women using illicit drugs. Providing antenatal care to such users from the early stages of pregnancy could prove to be beneficial in mitigating the associated adverse outcomes [76]. Currently, in Switzerland, opioid-dependent pregnant women are prescribed opioid agonist therapy like methadone in maintenance programs to reduce the risk of any adverse outcomes associated with opioid dependence [77, 78]. Nevertheless, these interventions could be further strengthened through interprofessional community-based programs that screen for those using illicit drugs in the early stages of pregnancy, provide targeted care and counseling on the ill effects of illicit drug use in pregnancy, and improve patient engagement in such care programs [76, 79]. Additionally, community-based services can also improve the collection of data on a comprehensive list of variables such as psychiatric conditions, psychoactive therapy, and other social and lifestyle factors associated with substance use in pregnancy. This would eventually facilitate better disentanglement of adverse effects due to substance use in pregnancy from potential confounders like psychoactive treatment in observational studies.

## Conclusion

In our study, consumption of cocaine and/or opioids during pregnancy was associated with an increased risk of adverse neonatal composite outcomes. We also observed a significant association between prenatal cocaine and/or opioid exposure and reduced odds of adverse obstetrical composite outcome. However, care should be taken in interpreting this result as it could be driven by the component on “induced labor” which is itself a consequence of various obstetrical and neonatal conditions. In this study, many infants of mothers reporting use of cocaine and/or opioids and simultaneously receiving psychoactive treatment during pregnancy were born preterm and/or had other adverse neonatal outcomes. Therefore, further studies with larger sample sizes are required to disentangle and elucidate the potential synergistic effect of psychoactive medication and illicit drugs in inducing adverse neonatal outcomes. Motivating those who use illicit drugs to seek antenatal care in the early stages of pregnancy and providing targeted therapy could potentially mitigate the adverse outcomes associated with illicit drug use in pregnancy. Implementing antenatal care programs by integrating several healthcare institutions in a community setting can encourage women to take up antenatal care early in pregnancy, improve patient engagement, and enable data collection for further research.

## Supplementary Information

Supplementary Material 1.

## Data Availability

The datasets generated and/or analyzed during the current study are not publicly available due to sensitive nature of the data and high risk of identifying individuals in our small study sample but are available from the corresponding author on reasonable request.

## Abbreviations

SGA: Small for gestational age
NAS: Neonatal abstinence syndrome
Apgar: Appearance, Pulse, Grimace, Activity and Respiration
Hep B: Hepatitis B
Hep C: Hepatitis C
SD: Standard deviation
IQR: Interquartile range
AOR: Adjusted odds ratio
CI: Confidence Interval
IPTW: Inverse probability treatment weighting
SMD: Standardized mean difference
OR_conditional_: Conditional Odds ratio
OR_crude_: Crude Odds ratio

## References

[1] Hussain CM, Rawtani D, Pandey G, Tharmavaram M. Chapter 7 - Raman spectroscopy in forensic science. In: Hussain CM, Rawtani D, Pandey G, Tharmavaram M, editors. Handbook of Analytical Techniques for Forensic Samples. Elsevier; 2021. p. 109–28.

[2] Substance Abuse and Mental Health Services Administration. Results from the 2012 National Survey on Drug Use and Health: Summary of National Findings [Internet]. [cited 2023 Apr 25]. Available from: https://www.samhsa.gov/data/sites/default/files/NSDUHnationalfindingresults2012/NSDUHnationalfindingresults2012/NSDUHresults2012.htm.

[3] Substance Abuse and Mental Health Services Administration. 2021 NSDUH Detailed Tables | CBHSQ Data [Internet]. [cited 2023 Apr 27]. Available from: https://www.samhsa.gov/data/report/2021-nsduh-detailed-tables.

[4] Tavella RA, de Abreu VOM, Muccillo-Baisch AL, da Silva Júnior FMR. Prevalence of illicit drug use during pregnancy: a global perspective. An Acad Bras Cienc. 2020;92(4):e20200302. 10.1590/0001-3765202020200302 33295578

[5] Addis A, Moretti ME, Ahmed Syed F, Einarson TR, Koren G. Fetal effects of cocaine: an updated meta-analysis. Reprod Toxicol. 2001;15(4):341–69. 11489591 10.1016/s0890-6238(01)00136-8

[6] Wendell AD. Overview and Epidemiology of Substance Abuse in Pregnancy. Clin Obstet Gynecol. 2013;56(1):91. 23314721 10.1097/GRF.0b013e31827feeb9

[7] Gouin K, Murphy K, Shah PS. Effects of cocaine use during pregnancy on low birthweight and preterm birth: systematic review and meta-analyses. Am J Obstet Gynecol. 2011;204(4):340.e1-340.e12. 21257143 10.1016/j.ajog.2010.11.013

[8] Minnes S, Lang A, Singer L. Prenatal Tobacco, Marijuana, Stimulant, and Opiate Exposure: Outcomes and Practice Implications. Addict Sci Clin Pract. 2011;6(1):57–70. 22003423 PMC3188826

[9] Little BB, Snell LM, Knoll KA, Ghali FE, Rosenfeld CR, Gant NF. Heroin abuse during pregnancy: Effects on perinatal outcome and early childhood growth. Am J Hum Biol Off J Hum Biol Counc. 1991;3(5):463–8. 10.1002/ajhb.1310030506 28597500

[10] Minozzi S, Amato L, Jahanfar S, Bellisario C, Ferri M, Davoli M. Maintenance agonist treatments for opiate-dependent pregnant women. Cochrane Database Syst Rev. 2020;2020(11):CD006318. 10.1002/14651858.CD006318.pub4 PMC8094273 33165953

[11] Kinsella M, Halliday LOE, Shaw M, Capel Y, Nelson SM, Kearns RJ. Buprenorphine Compared with Methadone in Pregnancy: A Systematic Review and Meta-Analysis. Subst Use Misuse. 2022;57(9):1400–16. 35758300 10.1080/10826084.2022.2083174

[12] Suarez EA, Huybrechts KF, Straub L, Hernández-Díaz S, Jones HE, Connery HS, et al. Buprenorphine versus Methadone for Opioid Use Disorder in Pregnancy. N Engl J Med. 2022;387(22):2033–44. 36449419 10.1056/NEJMoa2203318 PMC9873239

[13] Conner SN, Bedell V, Lipsey K, Macones GA, Cahill AG, Tuuli MG. Maternal Marijuana Use and Adverse Neonatal Outcomes: A Systematic Review and Meta-analysis. Obstet Gynecol. 2016;128(4):713–23. 27607879 10.1097/AOG.0000000000001649

[14] Gunn JKL, Rosales CB, Center KE, Nuñez A, Gibson SJ, Christ C, et al. Prenatal exposure to cannabis and maternal and child health outcomes: a systematic review and meta-analysis. BMJ Open. 2016;6(4):e009986. 27048634 10.1136/bmjopen-2015-009986 PMC4823436

[15] Marchand G, Masoud AT, Govindan M, Ware K, King A, Ruther S, et al. Birth Outcomes of Neonates Exposed to Marijuana in Utero. JAMA Netw Open. 2022;5(1): e2145653. 35084479 10.1001/jamanetworkopen.2021.45653 PMC8796018

[16] Volpe JJ. Effect of Cocaine Use on the Fetus. N Engl J Med. 1992;327(6):399–407. 1625714 10.1056/NEJM199208063270607

[17] Lind JN, Interrante JD, Ailes EC, Gilboa SM, Khan S, Frey MT, et al. Maternal Use of Opioids During Pregnancy and Congenital Malformations: A Systematic Review. Pediatrics. 2017;139(6): e20164131. 28562278 10.1542/peds.2016-4131 PMC5561453

[18] Graeve R, Balalian AA, Richter M, Kielstein H, Fink A, Martins SS, et al. Infants’ prenatal exposure to opioids and the association with birth outcomes: A systematic review and meta-analysis. Paediatr Perinat Epidemiol. 2022;36(1):125–43. 34755358 10.1111/ppe.12805

[19] Lutiger B, Graham K, Einarson TR, Koren G. Relationship between gestational cocaine use and pregnancy outcome: a meta-analysis. Teratology. 1991;44(4):405–14. 1835806 10.1002/tera.1420440407

[20] Santana DS, Silveira C, Costa ML, Souza RT, Surita FG, Souza JP, et al. Perinatal outcomes in twin pregnancies complicated by maternal morbidity: evidence from the WHO Multicountry Survey on Maternal and Newborn Health. BMC Pregnancy Childbirth. 2018;18(1):449. 30453908 10.1186/s12884-018-2082-9 PMC6245698

[21] Rizwan N, Abbasi RM, Mughal R. Maternal morbidity and perinatal outcome with twin pregnancy. J Ayub Med Coll Abbottabad JAMC. 2010;22(2):105–7. 21702280

[22] Battaglia FC, Lubchenco LO. A practical classification of newborn infants by weight and gestational age. J Pediatr. 1967;71(2):159–63. 6029463 10.1016/s0022-3476(67)80066-0

[23] Siu A, Robinson CA. Neonatal Abstinence Syndrome: Essentials for the Practitioner. J Pediatr Pharmacol Ther JPPT. 2014;19(3):147–55. 25309144 10.5863/1551-6776-19.3.147 PMC4187528

[24] Yadav S, Lee B, Kamity R. Neonatal Respiratory Distress Syndrome [Internet]. StatPearls [Internet]. StatPearls Publishing; 2022 [cited 2023 Mar 14]. Available from: https://www.ncbi.nlm.nih.gov/books/NBK560779/.

[25] Pinheiro RL, Areia AL, Mota Pinto A, Donato H. Advanced Maternal Age: Adverse Outcomes of Pregnancy. A Meta-Analysis Acta Med Port. 2019;32(3):219–26. 30946794 10.20344/amp.11057

[26] Behboudi-Gandevani S, Bidhendi-Yarandi R, Panahi MH, Mardani A, Paal P, Prinds C, et al. Adverse Pregnancy Outcomes and International Immigration Status: A Systematic Review and Meta-analysis. Ann Glob Health. 2022;88(1):44. 35854922 10.5334/aogh.3591 PMC9248985

[27] Shah PS, Zao J, Ali S, Knowledge Synthesis Group of Determinants of preterm/LBW births. Maternal marital status and birth outcomes: a systematic review and meta-analyses. Matern Child Health J. 2011;15(7):1097–109. 10.1007/s10995-010-0654-z 20690038

[28] Nelson HD, Darney BG, Ahrens K, Burgess A, Jungbauer RM, Cantor A, et al. Associations of Unintended Pregnancy With Maternal and Infant Health Outcomes: A Systematic Review and Meta-analysis. JAMA. 2022;328(17):1714–29. 36318133 10.1001/jama.2022.19097 PMC9627416

[29] Patra J, Bakker R, Irving H, Jaddoe V, Malini S, Rehm J. Dose–response relationship between alcohol consumption before and during pregnancy and the risks of low birthweight, preterm birth and small for gestational age (SGA)—a systematic review and meta-analyses. BJOG Int J Obstet Gynaecol. 2011;118(12):1411–21. 10.1111/j.1471-0528.2011.03050.x PMC3394156 21729235

[30] Nesari M, Olson JK, Vandermeer B, Slater L, Olson DM. Does a maternal history of abuse before pregnancy affect pregnancy outcomes? A systematic review with meta-analysis. BMC Pregnancy Childbirth. 2018;18(1):404. 30326858 10.1186/s12884-018-2030-8 PMC6192330

[31] Gedefaw G, Alemnew B, Demis A. Adverse fetal outcomes and its associated factors in Ethiopia: a systematic review and meta-analysis. BMC Pediatr. 2020;20(1):269. 32493464 10.1186/s12887-020-02176-9 PMC7268488

[32] Wondemagegn AT, Alebel A, Tesema C, Abie W. The effect of antenatal care follow-up on neonatal health outcomes: a systematic review and meta-analysis. Public Health Rev. 2018;39(1):33. 30574407 10.1186/s40985-018-0110-y PMC6296103

[33] Gold KJ, Marcus SM. Effect of maternal mental illness on pregnancy outcomes. Expert Rev Obstet Gynecol. 2008;3(3):391–401.

[34] Kautzky A, Slamanig R, Unger A, Höflich A. Neonatal outcome and adaption after in utero exposure to antidepressants: A systematic review and meta-analysis. Acta Psychiatr Scand. 2022;145(1):6–28. 34486740 10.1111/acps.13367

[35] Grigoriadis S, Graves L, Peer M, Mamisashvili L, Ruthirakuhan M, Chan P, et al. Pregnancy and Delivery Outcomes Following Benzodiazepine Exposure: A Systematic Review and Meta-analysis. Can J Psychiatry Rev Can Psychiatr. 2020;65(12):821–34. 10.1177/0706743720904860 PMC7658418 32148076

[36] Joseph-Delaffon K, Eletri L, Dechartres A, Nordeng HME, Richardson JL, Elefant E, et al. Neonatal outcomes after in utero exposure to antipsychotics: a systematic review and meta-analysis. Eur J Epidemiol. 2024;39(10):1073–96. 10.1007/s10654-024-01156-y 39352602

[37] Coughlin CG, Blackwell KA, Bartley C, Hay M, Yonkers KA, Bloch MH. Obstetric and Neonatal Outcomes After Antipsychotic Medication Exposure in Pregnancy. Obstet Gynecol. 2015;125(5):1224–35. 25932852 10.1097/AOG.0000000000000759 PMC4418034

[38] Reddick KLB, Jhaveri R, Gandhi M, James AH, Swamy GK. Pregnancy outcomes associated with viral hepatitis. J Viral Hepat. 2011;18(7):e394-398. 21692952 10.1111/j.1365-2893.2011.01436.x

[39] Yee LM, Kacanek D, Brightwell C, Haddad LB, Jao J, Powis KM, et al. Marijuana, Opioid, and Alcohol Use Among Pregnant and Postpartum Individuals Living With HIV in the US. JAMA Netw Open. 2021;4(12): e2137162. 34860242 10.1001/jamanetworkopen.2021.37162 PMC8642784

[40] Shinar S, Agrawal S, Ryu M, Walmsley S, Serghides L, Yudin MH, et al. Perinatal outcomes in women living with HIV-1 and receiving antiretroviral therapy-a systematic review and meta-analysis. Acta Obstet Gynecol Scand. 2022;101(2):168–82. 34704251 10.1111/aogs.14282 PMC9564575

[41] Atowoju I, Dawer P, Asrani M, Panjiyar B. Impact of maternal HIV infection on perinatal outcomes: A systematic review. Int J Gynecol Obstet. 2024;166(1):35–43. 10.1002/ijgo.15528 38573155

[42] Afraie M, Moradi G, Zamani K, Azami M, Moradi Y. The effect of hepatitis B virus on the risk of pregnancy outcomes: a systematic review and meta-analysis of cohort studies. Virol J. 2023;20(1):213. 37710321 10.1186/s12985-023-02182-0 PMC10500763

[43] Huang Qt, Huang Q, Zhong M, Wei Ss, Luo W, Li F, et al. Chronic hepatitis C virus infection is associated with increased risk of preterm birth: a meta-analysis of observational studies. J Viral Hepat. 2015;22(12):1033–42. 26081198 10.1111/jvh.12430

[44] Shen GF, Ge CH, Shen W, Liu YH, Huang XY. Association between hepatitis C infection during pregnancy with maternal and neonatal outcomes: a systematic review and meta-analysis. Eur Rev Med Pharmacol Sci. 2023;27(8):3475–88. 37140297 10.26355/eurrev_202304_32120

[45] Negrato CA, Mattar R, Gomes MB. Adverse pregnancy outcomes in women with diabetes. Diabetol Metab Syndr. 2012;4(1):41. 22964143 10.1186/1758-5996-4-41 PMC3514247

[46] Bramham K, Parnell B, Nelson-Piercy C, Seed PT, Poston L, Chappell LC. Chronic hypertension and pregnancy outcomes: systematic review and meta-analysis. BMJ. 2014;348: g2301. 24735917 10.1136/bmj.g2301 PMC3988319

[47] Viale L, Allotey J, Cheong-See F, Arroyo-Manzano D, Mccorry D, Bagary M, et al. Epilepsy in pregnancy and reproductive outcomes: a systematic review and meta-analysis. The Lancet. 2015;386(10006):1845–52. 10.1016/S0140-6736(15)00045-8 26318519

[48] Guo C, Wan M, Wang Y, Wang P, Tousey-Pfarrer M, Liu H, et al. Associations between intimate partner violence and adverse birth outcomes during pregnancy: a systematic review and meta-analysis. Front Med. 2023;10:1140787. 10.3389/fmed.2023.1140787 PMC10230039 37265489

[49] East CE, Biro MA, Fredericks S, Lau R. Support during pregnancy for women at increased risk of low birthweight babies. Cochrane database syst rev. 2023;4(4):CD000198. 10.1002/14651858.CD000198.pub3 PMC6443020 30933309

[50] Elsenbruch S, Benson S, Rücke M, Rose M, Dudenhausen J, Pincus-Knackstedt MK, et al. Social support during pregnancy: effects on maternal depressive symptoms, smoking and pregnancy outcome. Hum Reprod. 2007;22(3):869–77. 17110400 10.1093/humrep/del432

[51] Feldman PJ, Dunkel-Schetter C, Sandman CA, Wadhwa PD. Maternal Social Support Predicts Birth Weight and Fetal Growth in Human Pregnancy. Psychosom Med. 2000;62(5):715. 11020102 10.1097/00006842-200009000-00016

[52] Thomson K, Moffat M, Arisa O, Jesurasa A, Richmond C, Odeniyi A, et al. Socioeconomic inequalities and adverse pregnancy outcomes in the UK and Republic of Ireland: a systematic review and meta-analysis. BMJ Open. 2021;11(3): e042753. 33722867 10.1136/bmjopen-2020-042753 PMC7959237

[53] Bosworth OM, Padilla-Azain MC, Adgent MA, Spieker AJ, Wiese AD, Pham A, et al. Prescription Opioid Exposure During Pregnancy and Risk of Spontaneous Preterm Delivery. JAMA Netw Open. 2024;7(2): e2355990. 38353951 10.1001/jamanetworkopen.2023.55990 PMC10867678

[54] Yen E, Davis JM. The immediate and long-term effects of prenatal opioid exposure. Front Pediatr. 2022;10:1039055. 36419918 10.3389/fped.2022.1039055 PMC9676971

[55] Rosenfeld CS. The placenta as a target of opioid drugs. Biol Reprod. 2022;106(4):676. 35024817 10.1093/biolre/ioac003 PMC9040663

[56] Anbalagan S, Mendez MD. Neonatal Abstinence Syndrome. StatPearls. StatPearls Publishing; 2022 [cited 2022 Aug 23]. Available from: https://www.ncbi.nlm.nih.gov/books/NBK551498/. 31855342

[57] The American college of Obstetricians and Gynecologists. Committee Opinion No. 711: Opioid Use and Opioid Use Disorder in Pregnancy. Obstet Gynecol. 2017;130(2):e81-94. 28742676 10.1097/AOG.0000000000002235

[58] Zankl A, Martin J, Davey JG, Osborn DA. Opioid treatment for opioid withdrawal in newborn infants. Cochrane Database Syst Rev. 2021;7(7):CD002059. 10.1002/14651858.CD002059.pub4 PMC8261830 34231914

[59] Cleary BJ, Donnelly J, Strawbridge J, Gallagher PJ, Fahey T, Clarke M, et al. Methadone dose and neonatal abstinence syndrome-systematic review and meta-analysis: Methadone dose and neonatal abstinence syndrome. Addiction. 2010;105(12):2071–84. 20840198 10.1111/j.1360-0443.2010.03120.x

[60] Mayes LC, Carroll KM. Neonatal Withdrawal Syndrome in Infants Exposed to Cocaine and Methadone. Subst Use Misuse. 1996;31(2):241–53. 8834011 10.3109/10826089609045811

[61] Fulroth R, Phillips B, Durand DJ. Perinatal Outcome of Infants Exposed to Cocaine and/or Heroin In Utero. Am J Dis Child. 1989;143(8):905–10. 2756964 10.1001/archpedi.1989.02150200057018

[62] Noble LM, Hand IL, Hartstein J, Kim H, Yoon JJ. Cocaine Attenuates Neonatal Withdrawal in Methadone-Maintained Mothers† 1226. Pediatr Res. 1997;41(4):207–207.

[63] Huang H, Coleman S, Bridge JA, Yonkers K, Katon W. A meta-analysis of the relationship between antidepressant use in pregnancy and the risk of preterm birth and low birth weight. Gen Hosp Psychiatry. 2014;36(1):13–8. 24094568 10.1016/j.genhosppsych.2013.08.002 PMC3877723

[64] Grigoriadis S, Alibrahim A, Mansfield JK, Sullovey A, Robinson GE. Hypnotic benzodiazepine receptor agonist exposure during pregnancy and the risk of congenital malformations and other adverse pregnancy outcomes: A systematic review and meta-analysis. Acta Psychiatr Scand. 2022;146(4):312–24. 35488412 10.1111/acps.13441

[65] Grigoriadis S, VonderPorten EH, Mamisashvili L, Eady A, Tomlinson G, Dennis CL, et al. The effect of prenatal antidepressant exposure on neonatal adaptation: a systematic review and meta-analysis. J Clin Psychiatry. 2013;74(4):e309-320. 23656856 10.4088/JCP.12r07967

[66] Wang J, Cosci F. Neonatal Withdrawal Syndrome following Late in utero Exposure to Selective Serotonin Reuptake Inhibitors: A Systematic Review and Meta-Analysis of Observational Studies. Psychother Psychosom. 2021;90(5):299–307. 33971648 10.1159/000516031

[67] Källén B, Borg N, Reis M. The Use of Central Nervous System Active Drugs During Pregnancy. Pharmaceuticals. 2013;6(10):1221–86. 24275849 10.3390/ph6101221 PMC3817603

[68] Sanlorenzo LA, Cooper WO, Dudley JA, Stratton S, Maalouf FI, Patrick SW. Increased Severity of Neonatal Abstinence Syndrome Associated With Concomitant Antenatal Opioid and Benzodiazepine Exposure. Hosp Pediatr. 2019;9(8):569–75. 31262946 10.1542/hpeds.2018-0227 PMC6663519

[69] Leight KL, Fitelson EM, Weston CA, Wisner KL. Childbirth and mental disorders. Int Rev Psychiatry Abingdon Engl. 2010;22(5):453–71. 10.3109/09540261.2010.514600 PMC7061336 21047159

[70] Dunkel Schetter C, Tanner L. Anxiety, depression and stress in pregnancy: implications for mothers, children, research, and practice. Curr Opin Psychiatry. 2012;25(2):141–8. 22262028 10.1097/YCO.0b013e3283503680 PMC4447112

[71] Biaggi A, Conroy S, Pawlby S, Pariante CM. Identifying the women at risk of antenatal anxiety and depression: A systematic review. J Affect Disord. 2016;191:62–77. 26650969 10.1016/j.jad.2015.11.014 PMC4879174

[72] Pentecost R, Latendresse G, Smid M. Scoping Review of the Associations Between Perinatal Substance Use and Perinatal Depression and Anxiety. J Obstet Gynecol Neonatal Nurs JOGNN. 2021;50(4):382–91. 33773955 10.1016/j.jogn.2021.02.008 PMC8286297

[73] Cook JL, Green CR, de la Ronde S, Dell CA, Graves L, Morgan L, et al. Screening and Management of Substance Use in Pregnancy: A Review. J Obstet Gynaecol Can. 2017;39(10):897–905. 28935055 10.1016/j.jogc.2017.07.017

[74] Lester BM, Andreozzi L, Appiah L. Substance use during pregnancy: time for policy to catch up with research. Harm Reduct J. 2004;1(1):5. 15169566 10.1186/1477-7517-1-5 PMC419718

[75] Stone R. Pregnant women and substance use: fear, stigma, and barriers to care. Health Justice. 2015;3(1):2.

[76] Louw KA. Substance use in pregnancy: The medical challenge. Obstet Med. 2018;11(2):54–66. 29997687 10.1177/1753495X17750299 PMC6038015

[77] Kashiwagi M, Arlettaz R, Lauper U, Zimmermann R, Hebisch G. Methadone maintenance program in a Swiss perinatal center: (I): Management and outcome of 89 pregnancies. Acta Obstet Gynecol Scand. 2005;84(2):140–4. 15683373 10.1111/j.0001-6349.2005.00497.x

[78] Federal office of Public Health. Substitution-assisted treatments in case of opioid dependence. [cited 2024 Nov 11]. Available from: https://www.bag.admin.ch/bag/en/home/gesund-leben/sucht-und-gesundheit/suchtberatung-therapie/substitutionsgestuetzte-behandlung.html.

[79] Gulbransen K, Thiessen K, Ford N, Beck WP, Watson H, Gregory P. Interprofessional Care Models for Pregnant and Early-Parenting Persons Who Use Substances: A Scoping Review. Int J Integr Care. 2024;24(2):24. 38855026 10.5334/ijic.7589 PMC11160395

