# Supplementary material for: Risk of adverse obstetrical and neonatal outcomes in women consuming recreational drugs during pregnancy
Source: BMC Pregnancy Childbirth. 2025 Apr 16;25:456. doi: 10.1186/s12884-024-07062-1 (PMC12004786; doi:10.1186/s12884-024-07062-1)
Supplement: Supplementary file 1 — Supplementary Material 1. [file 12884_2024_7062_MOESM1_ESM.pdf]

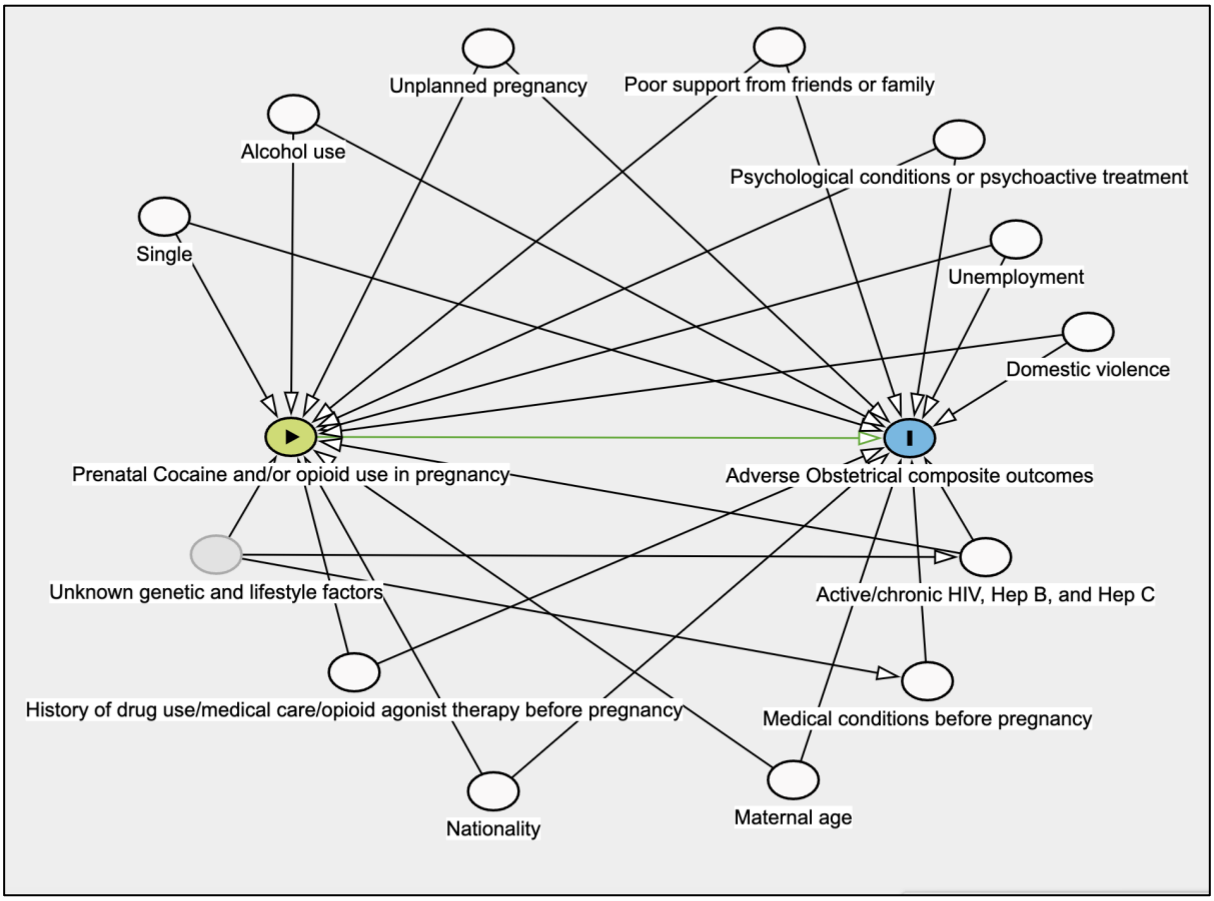

Figure S1: Potential confounders for Adverse obstetrical composite outcomes.

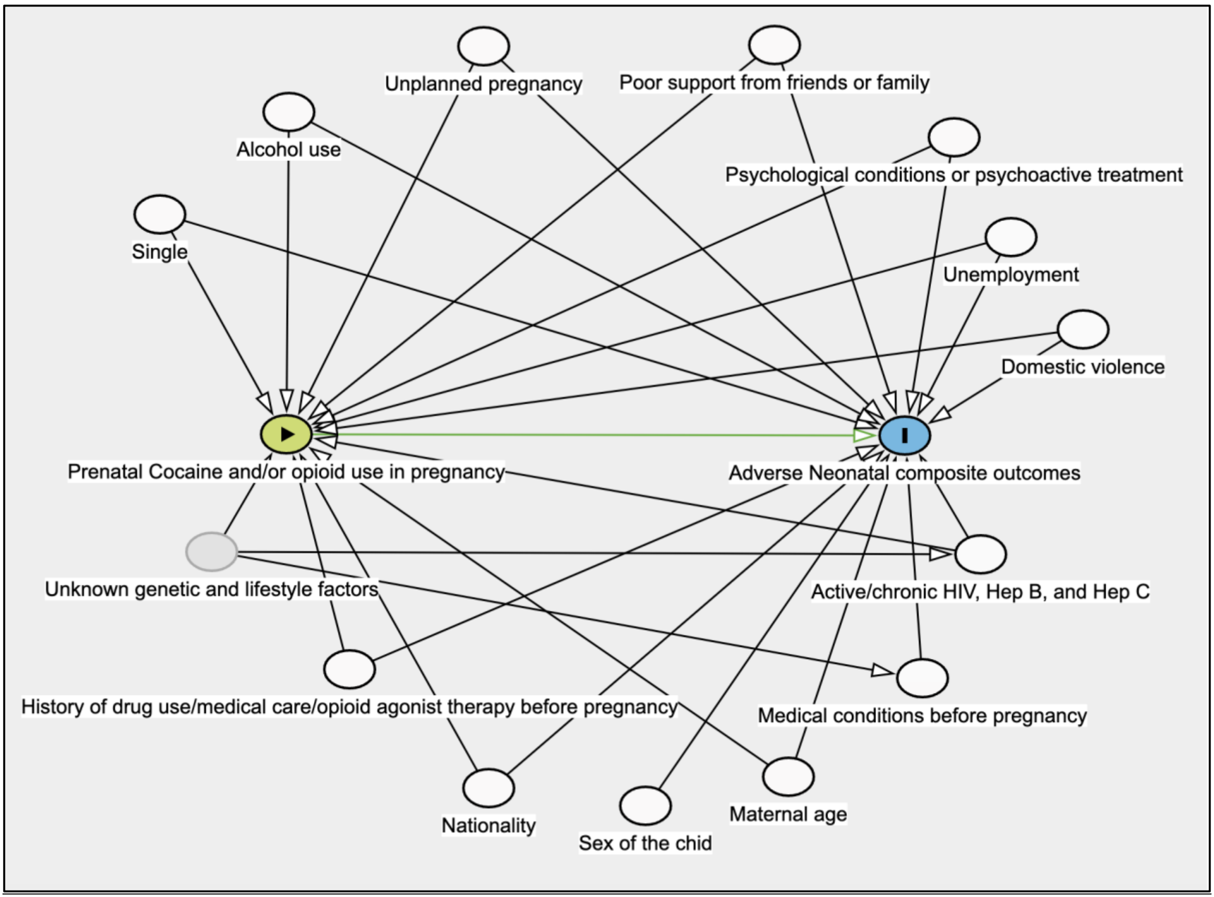

Figure S2: Potential confounders for Adverse Neonatal composite outcomes.
